# Chromosome-level genome assembly and annotation of *Anthurium amnicola*

**DOI:** 10.1038/s41597-025-04939-4

**Published:** 2025-04-10

**Authors:** Haomin Lyu, Sheina B. Sim, Scott M. Geib, Joanne S. L. Imamura, Briette L. Corpuz, Renee L. Corpuz, Angela N. Kauwe, Tyler J. Simmonds, Claire N. Arakawa, Roxana Y. Myers, Lisa M. Keith, Qingyi Yu, Tracie K. Matsumoto, Teresita D. Amore, Jon Y. Suzuki

**Affiliations:** 1 USDA ARS Daniel K. Inouye, U.S. Pacific Basin Agricultural Research Center, Hilo, Hawaii 96720 USA; 2 Hawaii Agriculture Research Center, Kunia, Hawaii 96759 USA; 3 Department of Tropical Plant and Soil Sciences, University of Hawaii at Manoa, Honolulu, Hawaii 96822 USA

**Keywords:** Plant genetics, Plant evolution, Agricultural genetics, Phylogenomics

## Abstract

*Anthurium amnicola* is in the monocot family Araceae, subfamily Pothoideae and is a contributing species in Hawaii floriculture industry hybrids. To support future genetic improvements to this commodity, we sequenced and assembled the *A. amnicola* genome to chromosome-scale using PacBio HiFi and short-read Hi-C sequencing. A total of 98.51% of the 4.79 Gb genome is anchored into 15 chromosomes, with 99.2% gene completeness and a high LTR assembly index (LAI) score of 21.73, indicative of a complete, high-quality assembly. Annotation reveals the presence of 20,380 protein-coding genes, with 78.52% of the genome composed of repetitive sequences, predominantly long terminal repeat retrotransposons (LTR-RT). Phylogenetic analysis identified evolutionary relationships between *A. amnicola* and representative species in the Araceae and other plant families. This study provides the first reference genome sequence for the neotropical genus *Anthurium* and insights into Araceae evolution, benefiting the floriculture industry and evolutionary studies.

## Background & Summary

**Fig. 1:**
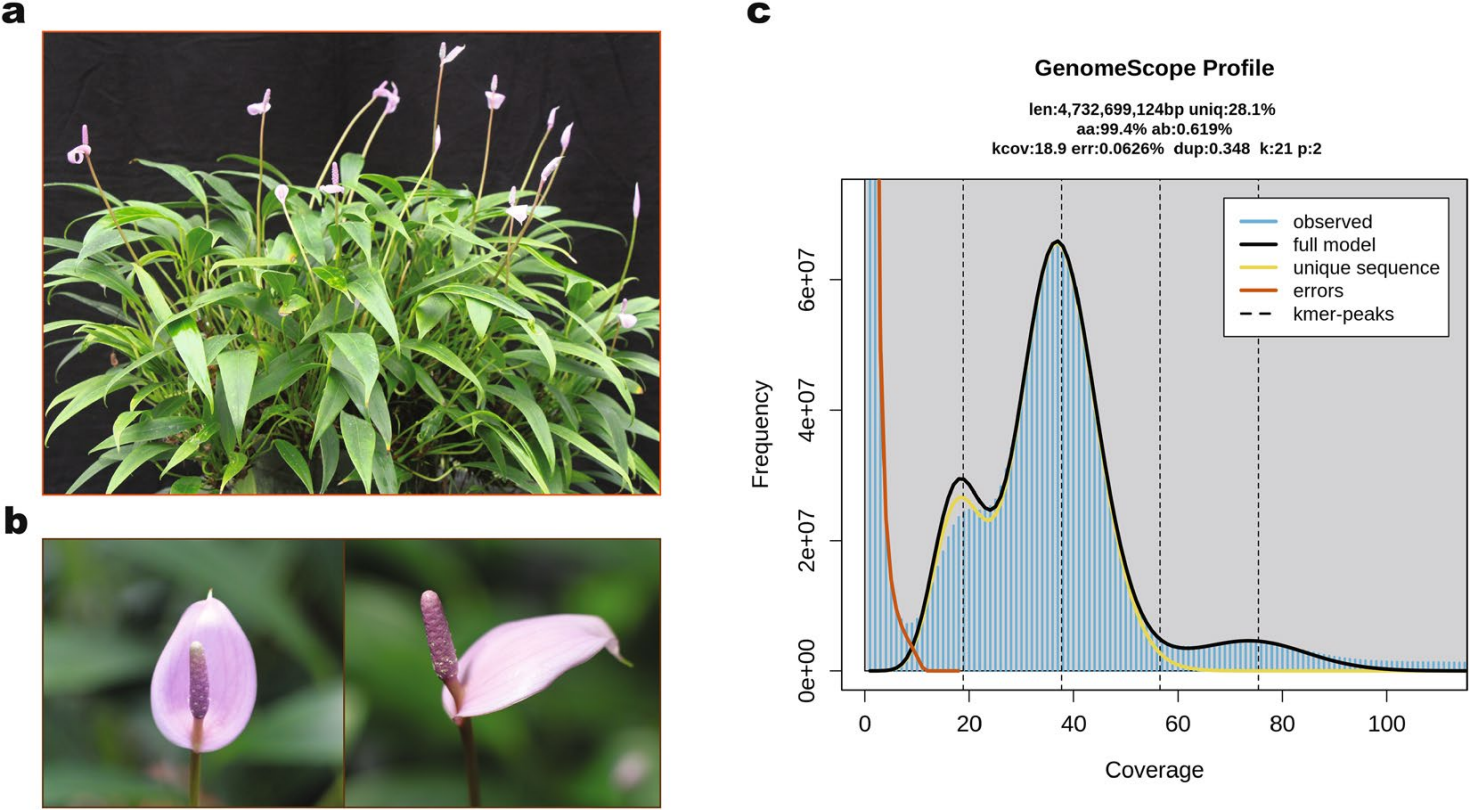
*Anthurium amnicola* Dressler, physical and genomic features. (**a**) Whole plant. (**b**) Floral stem (spathe and spadix). (**c**) GenomeScope2 analysis based on K-mer data indicating an estimation of genome size and heterozygosity.

*Anthurium amnicola*, first described in 1978, is a distinctive species belonging to the genus section *Calomystrium*, subsection *Rupicola*^1,2^. This species is endemic to the stream-side habitats of Panama, where it thrives in the unique ecological niches these environments provide^3,4^. The floral stem of this species consists of a spathe, a structure akin to a modified leaf and a spadix, an organ from which the female and male flowers arise; together these floral structures are characteristic of the family Araceae (Fig. 1a,b). These floral structures are not only visually striking but also serve as the basis for the species’ inclusion in ornamental breeding programs^5,6^.

The integration of *A. amnicola* by intergeneric hybridization has been instrumental in enhancing the aesthetic and horticultural traits of cultivated anthurium varieties. This effort has a rich history, particularly in Hawaii, where anthurium breeding has been a focal point for improving the crop’s commercial value and global appeal^7–11^. Research endeavors have spanned a range of disciplines, from genetics and the study of trait-associated genes to investigations into color^7,12–23^, physiology^24–28^, productivity^29^, form^30^, scent^31–33^, nematode susceptibility^34–38^ and bacterial blight susceptibility and resistance^39–42^, among others. Recent advancements in tissue culture propagation^43–48^ and biotechnology^49^ have further supported the commercial expansion of anthurium cultivation.

Nuclear and chloroplast DNA sequences have proven invaluable for elucidating phylogenetic relationships among species in *Anthurium*, the largest genus in the Araceae family, and for understanding its relationship with other family members^50–53^. Molecular marker sequences have also been useful for assessing the variedness and relationships of cultivars and species within germplasm collections^54–56^ and linkage maps of *Anthurium* have been produced^57,58^ for use in the mapping of genetic traits. However, until now a reference genome to clarify genome structure and content has been absent, limiting the depth of phylogenetic and evolutionary studies and breeding efforts.

Most commercial anthurium cultivars are complex hybrids or have uncertain lineage. *A. amnicola* was selected for whole-genome sequencing due to its close relation to commercial cultivars and its known genome size and chromosome number^5,59^. Unlike other species, *A. amnicola* produces numerous growing stems, from which flowers in sufficient quantities are produced for the study of floral traits^60^. Furthermore, previous studies on tissue-specific expression were anticipated to enhance gene annotation^16^.

To provide a high-quality reference genome for this valuable species, a chromosome-level genome of *A. amnicola* was generated through PacBio HiFi reads, coupled with the Hi-C technology for chromosome assembly. We determined a primary assembly of 4.79 Gb genome, aligning with prior estimates ranging from 4.765 Gb to 5.287 Gb for the haploid genome^59^ with a contig N50 of 26.98 Mb. The integration of Hi-C data with PacBio HiFi reads enabled the anchoring of 98.15% of the contig length into 15 pseudo-chromosomes, reflecting the cytological observation of a haploid chromosome count of 15 for *A. amnicola*^5^. The genome was annotated to include 20,380 protein-coding genes, with 78.52% of the genome attributed to transposable elements.

## Methods

### Sample collection, library construction and sequencing

A spathe from the floral stem of *A. amnicola* accession UH A667 was divided into four 50 mg samples (Fig. 1a,b). Samples were flash frozen in liquid nitrogen and processed on a MP FastPrep-24 classic (MP Biomedicals, Irvine, California, USA) at 4.5 m/s for 20 seconds in a 2 mL disruption tube (MP FastPrep Tubes) containing a metal grinding bead (BC Percision 5/32” 440 Stainless Steel Ball Bearings G100) and DNA was isolated using the PacBio Nanobind® Plant Nuclei Kit TR (Pacific Biosciences, Menlo Park, California, USA) (HMW DNA extraction from plant nuclei) following the manufacturers’ instructions. A whole genome sequencing PacBio HiFi library was prepared using the SMRTbell® prep kit 3.0 (Pacific Biosciences, Menlo Park, California, USA) following the manufacturer’s instructions. The final library was size selected using 35% v/v dilution of AMPure PB beads following the manufacturer’s instructions to remove fragments shorter than 5 kb. The library sample was quantified using the Invitrogen Qubit dsDNA HS Assay Kit, and the size distribution of the library was measured and visualized using the Agilent Femto Pulse System (Agilent Technologies, Santa Clara, CA, USA).

The HiFi library was then sequenced using the PacBio Revio System, obtaining a total of 181.0 Gb HiFi reads, representing approximately 40 × coverage of the *A. amnicola* genome. We performed GenomeScope2 (v2.0.1) analysis on the HiFi reads^61^, leading to an estimation of a genome size of 4.73 Gb and heterozygosity of 0.62% (Fig. 1c).

For the Hi-C dataset, tissue was cross-linked, digested using the enzymes DdeI and DpnII, biotinylated, and proximity ligated. After proximity ligation and nucleic acid purification, DNA was sheared using the Diagenode Bioruptor Pico, enriched for proximity ligations using streptavidin beads, and size-selected to enrich for biotin labeled DNA fragments 200–600 bp. Following fragmentation and sample purification using a room temperature polyethylene glycol solution containing solid-phase reversible immobilization (SPRI) beads^62^, library preparation was performed using the NEB Next Ultra II DNA Library Prep Kit and followed by the Element Biosciences Adept Library Prep Kit. The resulting Hi-C library was sequenced on a partial 2 × 150 bp flow cell of an Element Biosciences Aviti system (Element Biosciences, San Diego, California, USA).

### *De novo* assembly and Hi-C scaffolding

The PacBio HiFi reads were assembled using the Hifiasm (v0.19.5-r587)^63^ with the default parameters. Duplicate haplotigs and erroneous assemblies were removed using the Purge_Dup (v1.2.6) pipeline^64^. Raw Hi-C reads were aligned to the contig-level genome assembly using BWA-mem (v2.2.1)^65^, with subsequent removal of PCR-derived duplicates. The program, YAHS (v1.2a.1)^66^ was then used for scaffolding with default parameters. Assembly errors were manually adjusted using Juicebox (v1.11.08)^67^, culminating in the resolution of 15 chromosomes.

**Fig. 2:**
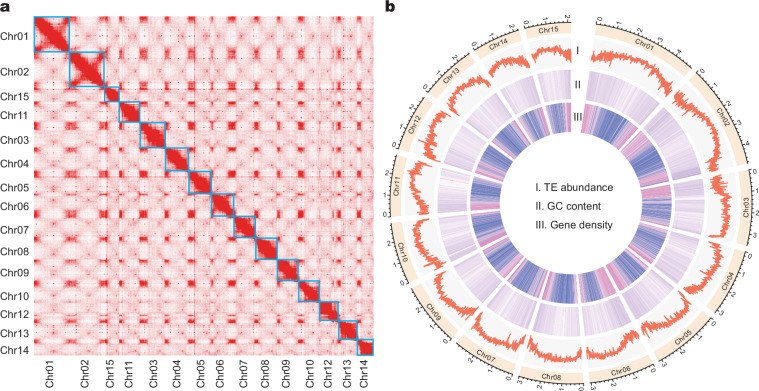
Chromosome-level assembly and characteristics of the *Anthurium amnicola* genome. (**a**) Hi-C chromosomal scaffolding heatmap. (**b**) The landscape of the *A. amnicola* genome. The circus plot depicts the 15 identified chromosomes and their genomic features: (I) abundance of transposable elements, (II) GC content, and (III) gene density.

The total length of the contig-level genome was 4.79 Gb (Table 1), approximately matching the genome size estimated by the flow cytometry method for this species, ranging from 4.77 to 5.29 Gb^59^. The assembly comprised 1,392 contigs, achieving an L50 of 26.98 Mb and an N50 of 56. After scaffolding, a total of 4.72 Gb of the assembled contigs were anchored into 15 pseudo-chromosomes, spanning 98.51% of the final genome assembly (Fig. 2a,b; Table 1).

**Table 1:** Statistics of the *Anthurium amnicola* genome assembly and scaffolding.

Assembly characteristics	Values
Main genome scaffold total number	984
Main genome scaffold sequence total length	4789.83 Mb
Main genome scaffold N50	6
Main genome scaffold L50	306.43 Mb
Main genome contig N50	56
Main genome contig L50	26.98 Mb
BUSCO	99.2%
LAI	21.73

### Annotation of repetitive sequences

To delineate the repetitive sequences across the *A. amnicola* genome, the EDTA pipeline for transposon prediction and annotation was employed using default parameters^68^. This pipeline combined three types based on their underlying approach: homology-based, structural-based, and *de novo* methods. Our analysis revealed a total of 3.76 Gb of repetitive sequences, constituting 78.52% of the *A. amnicola* genome assembly (Fig. 2b; Table 2). The predominant class was long terminal repeat retrotransposons (LTR-RT), which made up 82.06% of the total repetitive sequences (Table 2). DNA transposons were also identified, comprising 2.99% of the entire genome (Table 2).

**Table 2:** Information on the repeat annotation in the *Anthurium amnicola* genome assembly.

Class		bpMasked	%masked
LTR	Copia	375732402	7.84%
	Gypsy	1793785252	37.43%
	unknown	918175271	19.16%
TIR	CACTA	136682265	2.85%
	Mutator	113434220	2.37%
	PIF_Harbinger	35238676	0.74%
	Tc1_Mariner	12498453	0.26%
	hAT	64752156	1.35%
Non-LTR	LINE_element	9991839	0.21%
	unknown	404539	0.01%
Non-TIR	helitron	76003625	1.59%
repeat_region		225997332	4.72%
Total		3762696030	78.52%

### Prediction of gene structure and functional annotation

The protein-coding genes of the *A. amnicola* genome were predicted using a combination of three methods: *ab initio* gene prediction, homology-based gene prediction, and RNA-seq data gene prediction. Initially, RepeatMasker (v4.1.0)^69^ was applied to annotate the genome-wide transposons, providing the masked genome sequences for gene annotation. For homology-based predictions, we used GeMoMa (v1.9)^70^, aligning protein sequences from *Spirodela polyrhiza*^71^, *Lemna minor*^72^, *Zantedeschia elliottiana*^73^, and *Colocasia esculenta*^74^ to our genome assemblies. These four genomes exhibited high assembly quality and were publicly available to enable genome-wide comparisons, and thus chosen to represent main lineages of the Araceae (Table 3). Coding genes were predicted with GeMoMa (v1.9) using^70^ default parameters. Transcriptome evidence was derived from genome-guided assembly of RNA-seq data accessible in the NCBI database (PRJNA288827)^16^. Hisat2 (v2.2.0)^75^ was used to align the RNA-seq reads to the reference genomes, and TACO (v0.7.3)^76^ was employed to reconstruct the full-length transcripts, and Transdecoder (v5.7.0) (https://github.com/TransDecoder/TransDecoder) identified the candidate coding regions. Augustus (v3.3.2)^77^, GeneMark-ET (v4.59)^78^ and Braker2 (v2.1.2)^79^ were used to perform *ab initio* gene prediction with training based on mapping files of the RNA-seq data. Finally, EVidenceModeler (v2.0.0)^80^ was employed to integrate results from all three methods to produce a final gene prediction model. After filtering out low quality predictions, a total of 20,380 predicted protein-coding genes in the *A. amnicola* genome was obtained (Fig. 2b).

**Table 3:** Genomic information of select Araceae species.

	*A. amnicola*	*C. esculenta*	*L. minor*	*S. polyrhiza*	*Z. elliottiana*
Subfamily	Pothoideae	Aroideae	Lemnoideae	Lemnoideae	Philodendroideae
Genome size	4.79 Gb	2.41 Gb	472 Mb	158 Mb	1.15 Gb
Karyotype	2n = 2x = 30	2n = 2x = 28	2n = 2x = 40	2n = 2x = 64	2n = 2x = 32
Gene number	20,380	28,695	22,382	19,623	36,165
Repeat	78.52%	88.43%	61.50%	15.79%	60.18%

Functional annotation was conducted using eggNOG-mapper (v5.0)^81^. The protein-coding genes were used to compare with the eggNOG database and generated functional annotations for gene sets of *A. amnicola*. According to the results, the Gene Ontology (GO) and Kyoto Encyclopedia of Genes and Genomes (KEGG) pathways were assigned to 7,490 and 7,122 genes, respectively.

### Phylogenetic tree construction

To trace the evolutionary relationships among Alismatales species, we collected 19 high-quality genomes from public databases, including Phytozome. Our dataset comprised eight Araceae species, five non-Araceae Alismatales species, and five additional eudicots and monocots, with *Nymphaea colorata* serving as the outgroup^71–74,82–84^. The gene families across these 19 genomes were inferred using the OrthoFinder program (v2.5.5) with default parameters^85^. The entire gene set from all 19 genomes were compared with the publicly available databases of Swiss-Prot and RefSeq non-redundant proteins using BLASTP program, and against the Dfam database using HMMER (v3.4)^86,87^. When necessary, transposon protein genes could be removed from the gene set of any one of the 19 genomes in cases where they were not already removed prior to publication. Finally, the 20,380 protein-coding genes of *A. amnicola*, as well as those of the other 18 species, were assigned to 19,543 families, of which 305 were identified as single-copy gene families across all 19 species.

The genes representing the 305 single-copy gene families were then used to reconstruct the phylogenetic relationship among these species. First, the CDS sequences in each family were aligned based on protein sequence alignment using the program MUSCLE (v5.1.0) with default parameters^88^. The jmodeltest2 (v2.1.10) was then used to detect the best substitution model for the 305 genes across 19 species^89^, determining the optimal model of “GTR + I + G”. Subsequently, RAxML (v8.1.12) software was used to infer the phylogenetic tree with the parameters “-m GTRGAMMAI -f a” setting 1000 bootstraps and *N. colorata* as an outgroup^90^.

**Fig. 3:**
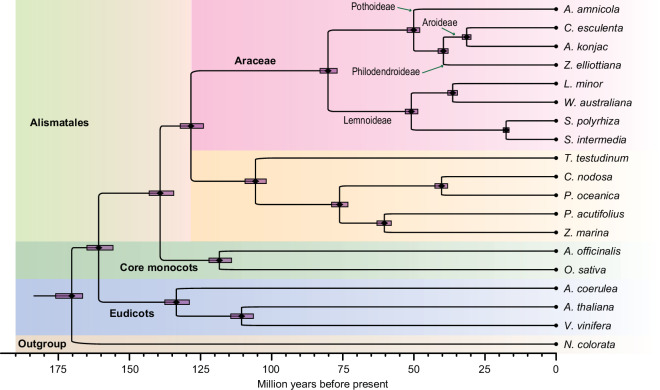
Phylogenetic relationships across select species in the monocotyledon order Alismatales. The genomes of *Anthurium amnicola* and 18 other species were used to infer the origin and divergent ages of Alismatales species including those representing four subfamilies of the family Araceae. The boxes at each node represent 95% confidence intervals.

Finally, the resulting phylogenetic relationship data together with the 305 gene sequences were used to infer the divergent time across the tree using MCMCtree implemented in PAML (v4.10.5)^91^. The divergent ages were calibrated using two time points, including the ancestor of *N. colorata* at 168~192 million years ago (MYA) and the divergent time between monocots and eudicots at 142~164 MYA^92^. Our analysis revealed the divergence age among different lineages of Alismatales (Fig. 3). Within the Araceae family, the aquatic lineage of Lemnoideae originated around 80 MYA, while the first investigated subfamily Pothoideae, represented by *A. amnicola*, diverged around 50 MYA (Fig. 3).

## Data Records

The PacBio HiFi long reads and Hi-C reads were deposited in the National Center for Biotechnology Information (NCBI) Sequence Read Archive (SRA) database under the SRA accession number SRP525474^93^. The genome assembly has been deposited in GenBank with the accessions GCA_041870085.1^94^. The genome annotation results have been deposited in the figshare database^95^.

## Technical Validation

The genome assembly quality was first evaluated using BUSCO (v5.2.2) and the orthologous database of embryophyta_odb10 (https://busco-data.ezlab.org/v5/data/lineages/), representing 1,614 single-copy genes conserved in embryophytes^96^. The BUSCO analysis revealed that the *A. amnicola* genome assembly achieved a completeness of 99.2% at the chromosome level, with 86.3% being single-copy and 12.9% duplicated genes (Table 1). This assessment indicated a highly complete genome assembly of *A. amnicola* in this study.

The completeness of genome assembly was also assessed employing the LTR Assembly Index (LAI)^97^, implemented in the LTR_retriever pipeline (v3.0.1)^98^. Following the detection of LTR-RT candidate elements using LTR_finder (v.1.5.1045) and LTR_harvest (v1.0646)^99,100^, the LTR_retriever pipeline was applied to filter the confidential LTR-RTs, and assessing genome assembly quality. These analyses showed a general LAI value of 21.73, indicative of a high continuity of the chromosomal genome assembly for *A. amnicola* (Table 1).

## Data Availability

All software and pipelines were executed according to the manual and protocols of the published bioinformatics tools. The version and parameters of the software have been described in Methods. The pipelines for genome assembly and gene annotation were accessible in the GitHub repository (https://github.com/HaominLyu/HiGenAA).
